# Longitudinal Associations Between Parental Bonding, Parenting Stress, and Executive Functioning in Toddlerhood

**DOI:** 10.1007/s10826-017-0679-7

**Published:** 2017-02-27

**Authors:** Evi S. A. de Cock, Jens Henrichs, Theo A. Klimstra, A. Janneke B. M. Maas, Charlotte M. J. M. Vreeswijk, Wim H. J. Meeus, Hedwig J. A. van Bakel

**Affiliations:** 1 Department of Developmental Psychology, Faculty of Social and Behavioral Sciences, Tilburg University, Tilburg, the Netherlands; 2 Department of Midwifery Science, AVAG and the EMGO+ Institute for Health and Care Research, VU University Medical Center, Amsterdam, the Netherlands; 3 FIOM, ’s Hertogenbosch, the Netherlands; 4 Apanta, GGZ, Veldhoven, the Netherlands; 5 Adolescent Development, Utrecht University, Utrecht, the Netherlands; 6 Centre for Child and Adolescent Psychiatry, Rosmalen, the Netherlands; 7 Tranzo, Faculty of Social and Behavioral Sciences, Tilburg University, Tilburg, the Netherlands

**Keywords:** Maternal bonding, Paternal bonding, Prenatal bonding, Parenting stress, Executive functioning

## Abstract

Early executive functioning is an important predictor for future development of children’s cognitive skills and behavioral outcomes. Parenting behavior has proven to be a key environmental determinant of child executive functioning. However, the association of parental affect and cognitions directed to the child with child executive functioning has been understudied. Therefore, in the present study we examine the associations between parental bonding (i.e., the affective tie from parent to child), parenting stress, and child executive functioning. At 26 weeks of pregnancy, and at 6 months and 24 months postpartum the quality of the maternal (N = 335) and paternal (N = 261) bond with the infant was assessed. At 24 months, postnatal parenting stress and child executive functioning were measured by means of parent-report questionnaires. Results indicated that for both mothers and fathers feelings of bonding negatively predicted experienced parenting stress over time. In addition, for both parents a negative indirect effect of bonding on child executive functioning problems was found via experienced parenting stress. These findings indicate the importance of monitoring parents who experience a low level and quality of early parent-child bonding, as this makes them vulnerable to parenting stress, consequently putting their children at risk for developing executive functioning problems.

## Introduction

In the 1st years of life, children are largely dependent on their caregivers, who primarily provide a child’s environmental context in this period. Particularly neurodevelopment is an ongoing and remarkably rapid process during the first 2 years of life (e.g., Fox et al. 1994; Knickmeyer et al. 2008) making the young child’s developing brain sensitive to environmental influences, such as early caregiving. Previous research suggests that early caregiving may significantly affect child neurodevelopmental outcomes (i.e., child (neuro)cognitive and behavioral outcomes; Bernier et al. 2010). A suboptimal parental bond (i.e., the affective tie from parent to child) not only negatively affects later child outcomes but is also detrimental for parents themselves. For example, studies have shown that poorer parental bonding is associated with higher levels of parenting stress (de Cock et al. 2016; Mason et al. 2011).

Early executive functioning is a key child cognitive outcome and an important precursor for future development of children’s cognitive skills and behavioral outcomes. Executive functioning is an umbrella term for higher-order neurocognitive processes that organize and direct cognition, emotion, and behavior (e.g., Garon et al. 2008; Isquith et al. 2005). These can be assessed as early as in infancy, and research shows that early individual differences in executive functioning are relatively stable over time (Carlson et al. 2004). When basic executive functions are sub optimally developed in toddlerhood and the preschool years, this negatively affects the development of more complex executive functioning, because the emergence of these higher-order skills is dependent on the achievement of simpler abilities. In this context, previous studies have shown that preschool executive functioning skills, such as inhibitory control and working memory, are predictive of school achievement (e.g., mathematical and reading abilities) in the 1st years of primary school as well as later in childhood (Blair and Razza 2007; Clark et al. 2010). Poor preschool executive functioning has also been linked to childhood internalizing and externalizing problem behavior (e.g., Roman et al. 2016; Sulik et al. 2015). Early executive functioning has a substantial impact on several important indicators of child psychosocial well-being and neurodevelopmental functioning, making it crucial to examine child executive functioning and its determinants early in life.

Executive functioning is primarily associated with brain activation in prefrontal regions, which show prolonged development and rapid growth during the first 2 years of life (e.g., Anderson et al. 2008; Diamond 2002). In this period, environmental factors such as parental behavior have a major impact on future child behaviors through their impact on the developing brain. Caregiving behavior fosters the development of children’s self-regulatory or executive functioning skills by providing the child with a predictive, orderly, and stimulating environment (Carlson 2003; Schroeder and Kelley 2010). According to Vygotsky’s theory (1978), children learn to structure thinking processes from interpersonal interaction, most likely with their parents, before these become internal and they can manage individually without help. In addition, through social (i.e., parent-infant) interactions, elementary cognitive processes develop into more sophisticated higher-order cognitive processes. Thus, early social relationships and caregiving behavior are important for children’s development of executive functioning skills.

Despite the fact that parental bonding is a key component of the caregiving relationship, this concept is still understudied. Parental bonding can be defined as a subjective experience of affection of the parent towards the child. The core of the parental bond is a feeling state (“love”) that eventually exposes itself in parental behavior (e.g., Condon 1993; Condon and Corkindale 1998). According to Condon’s theory, this process of parental feelings eventually expressing itself in behavior is facilitated by parents’ goal directed needs or dispositions to act (i.e. knowing, being with, avoiding separation and loss from, protecting, and gratifying needs of the child.). These dispositions can be considered indicators of parental bonding and are assessable through self-report. Already during pregnancy parental feelings, cognitions, and behavior towards the fetus begin to develop and can be validly and reliably assessed (e.g., Condon 1993). This is referred to as the *prenatal parental bond* (or maternal fetal attachment) and this unique feature of parental bonding makes it distinct from, for example, the infant-mother attachment relationship which focuses on attachment behavior of the infant (Walsh 2010).

Previous longitudinal research shows that the prenatal parental bond is strongly related to the postnatal bond and the quality of parent-infant interaction (Damato 2004; de Cock et al. 2016; Maas et al. 2016; Müller 1996). Mothers with higher levels of prenatal bonding display more parental involvement, and more sensitivity and stimulation when interacting with their child in the 1st year after birth (Siddiqui and Hägglöf 2000). Besides research on the association of bonding with actual parenting behavior, previous research also shows that parental feelings of bonding are associated with child outcomes. A lower quality of the postnatal bond, for example, has been found to be related to poorer later child social-emotional development and more difficult temperament (de Cock et al. 2016; Mason et al. 2011).

Although parental bonding seems to be an important predictor of child outcomes, the association with child executive functioning has not yet been investigated. Previous research on parental predictors of child executive functioning has focused on parenting *behaviors* instead of parental *feelings and cognitions*. These studies found that more parental scaffolding or autonomy support, more parental sensitivity, more parental stimulation, and less parental control/discipline are to a greater or lesser degree related to better child executive functioning (for a review see Fay‐Stammbach et al. 2014). In these previous studies, different parenting and caregiving dimensions have been examined with different effects on child executive functioning, indicating that parents have a significant impact on their children’s cognitive development in multiple ways. However, parental bonding, a potentially important cognitive and affective parental factor with a strong impact on parental behavior that can already be measured in the prenatal period, has not yet been studied in this context.

A poor parental bond not only has detrimental effects on child outcomes, but also the parents themselves are affected. For some parents developing a bond with their child does not come naturally or optimally. Disturbances in forming a parental bond can be stressful for parents and can have a negative effect on parenting abilities and feelings of parental adjustment (Müller 1994; Siddiqui and Hägglöf 2000). Previous research, using a person-centered method, showed that a pattern of low bonding levels from pregnancy to toddlerhood is related to more parenting stress (de Cock et al. 2016). In addition, studies found that maternal bonding has a buffering effect in the association between postpartum depression and parenting stress (Mason et al. 2011; Reck et al. 2016). In these studies, also a direct association between maternal bonding and parenting stress was found in the early postnatal period. Although most parents of young children will experience some parenting daily hassles, parents who lack the feeling of a strong bond with their child might experience a higher degree of parenting stress.

Similar to parental bonding, parenting stress has an effect on both the quality of parent-infant interaction and child outcomes. In a longitudinal study, Crnic et al. (2005) found that cumulative early parenting stress negatively affects future parental behavior and the quality of parent-infant interaction. In the same study, higher levels of parenting stress were also found to be related to more child behavior problems at 5 years of age. Moreover, parenting behavior did not mediate this association, indicating a direct effect of parenting stress on child outcomes (Crnic et al. 2005). In addition, studies have shown that parenting stress is related to child cognitive development and executive functioning. For example, more parenting stress at 6 months has a negative effect on child cognitive development (e.g. perception, problem solving, language) at 12 months (Molfese et al. 2010). Moreover, in a cross-sectional study parenting stress has also been negatively linked to child executive functioning at 8–12 years (Joyner et al. 2009). Taken together, the findings of the abovementioned studies suggest a possible indirect effect of parental feelings of bonding on child executive functioning through parenting stress.

Finally, most of the abovementioned studies only included mothers. Fathers are still underrepresented in research on parenting and the parent-infant relationship, while effects of father involvement on child developmental outcomes are significant and have been documented repeatedly (Ramchandani et al. 2013; Sarkadi et al. 2008). Therefore, the role of fathers regarding the development of child executive functioning is important to examine. A study by Lucassen et al. (2015) shows distinct effects of maternal and paternal parenting on child executive functioning. The authors found that less maternal, but not paternal sensitivity, and harsher parenting by fathers, but not by mothers, was related to lower scores on child executive functioning at 3 years. Another study found that both mothers’ as well as fathers’ sensitive and supportive parenting predicted children’s executive functioning at 3 years (Towe-Goodman et al. 2014). However, in most studies examining the effect of parenting on child executive functioning, maternal and paternal parenting effects are not studied separately. Rather they are analyzed together as one factor, which makes it impossible to observe a potential difference in effects. In addition, fathers tend to experience lower levels of bonding compared to mothers, and correlations between maternal and paternal bonding are only weak to moderate (de Cock et al. 2016; Hoffenkamp et al. 2012). Therefore, it is important to include fathers when studying the effects of parental variables on child outcomes and to examine paternal effects separately from maternal effects.

Findings from previous research raise the question to what extent bonding is directly related to child executive functioning and to what extent this association might be mediated by parenting stress. We examine this by means of the following two research questions. First, we examine the association of pre- and postnatal bonding with parenting stress at 24 months in both mothers and fathers. We hypothesize that parental (prenatal) bonding levels are positively related to successive bonding levels and that parental bonding is negatively related to the experience of parenting stress. Research on parental bonding and parenting stress beyond the early postnatal period is lacking, despite the fact that toddlerhood (or “terrible two’s”) is an important and well-known stressful parenting period in which children put their parents to the test due to their increasing autonomy (e.g., Crnic and Booth 1991). Second, we examine the association between early maternal and paternal bonding and child executive functioning in toddlerhood and the mediating role of parenting stress. We expect that parents who report lower levels of bonding, experience more parenting stress, and this in turn leads to more executive functioning problems in children at 2 years. Additionally, as previous research has demonstrated paternal influences on child executive functioning, we expect to find associations between parental bonding, parenting stress and child executive functioning for both mothers and fathers. By assessing executive functioning early in life, potential problems can be discovered in time which leaves more room for intervention. In addition, examining the determinants of early executive functioning also provides an opportunity to discover and monitor families at risk and children vulnerable for developing problems in their executive functioning abilities.

## Method

### Participants

Only parents of whom data was available on at least two measurement moments were included in the analyses. This resulted in a final sample of 335 mothers and 261 fathers and their children (81.9 % of the mothers and 81.8 % fathers who originally agreed to participate). Little’s Missing Completely At Random test (1988) revealed a normed Chi-Square (χ2/*df*) of 1.85 and 0.44 for mother and father data, respectively. This indicates a good fit between sample scores with and without imputation according to guidelines by Bollen (1989). Therefore, missing data were imputed with multiple imputation generating 10 complete datasets for conducting analyses in IBM SPSS Statistics 22. At baseline (i.e., 26 weeks of pregnancy mothers and fathers were on average 31.7 (SD = 4.23, range 17–43) and 34.2 (SD = 4.47, range 23–50) years old. Most parents were highly educated, as 65.4 % of the mothers and 67.3 % of the fathers had 9 or more years of education after primary school. Furthermore, 83.2 % of the mothers and 85.0 % of the fathers were Dutch. Children (49.6 % boys, 55.8 % firstborns) were born at a mean gestational age of 39.8 (SD = 1.6) weeks and a mean birth weight of 3427.8 (SD = 538.5) grams. All parents signed informed consent before participating in the study. This study has been approved by the medical ethics committee of the St. Elisabeth hospital in Tilburg, the Netherlands (date: 13-08-2008, registration number: NL 23376.008.08).

### Procedure

The present study is embedded in the prospective longitudinal cohort study ‘Expectant Parents’, which focuses on prenatal (risk) factors of postnatal child development, parenting, and parent-infant relationships. Detailed information regarding design and procedure of the study has been described elsewhere (Maas et al. 2012). In the current study, maternal and paternal bonding were assessed via postal questionnaires at 26 weeks of pregnancy, and at 6 months and 24 months postpartum. At 24 months, parents completed a questionnaire about parenting stress. Mothers also filled in a questionnaire about child executive functioning problems. Portions of the data used in the current study have been published before (de Cock et al. 2016). In this previous study, parental pre- and postnatal bonding was studied in association with multiple correlates in the parent- child- and contextual domain. However, parental bonding was studied by means of person-centered methods, which provided (four) patterns of bonding, instead of separate pre-and postnatal bonding variables as examined in the current study.

### Measures

#### Parental bonding

At 26 weeks of gestation, and at 6 and 24 months postpartum parents completed questionnaires about their feelings of parental bonding. To assess prenatal bonding, the Maternal and Paternal version of the Antenatal Attachment Scales were used (MAAS/PAAS; Condon 1993). The MAAS consists of 19 items which are scored 1–5, with higher scores indicating higher levels of bonding. As the PAAS consists of 16 items (also scored 1–5) the sum score was divided by 16 and multiplied by 19 to obtain scores that are comparable to the maternal bonding sum scores based on a 19-item bonding questionnaire. An example item of the MAAS/PAAS is “Over the past 2 weeks I have thought about, or have been preoccupied with the baby inside me/the developing baby”. Internal consistency of the MAAS and PAAS in the present study is sufficient (Cronbach’s alphas of .76 and .78, respectively).

For postnatal bonding, we used the Maternal and Paternal version of the Postnatal Attachment Scales (MPAS/PPAS; Condon and Corkindale 1998; Condon et al. 2008). Both scales consist of 19 items which are scored 1–5, with higher scores indicating higher levels of bonding. An example item for both the MPAS and PPAS is “When I am not with the baby/child, I find myself thinking about the baby/child”. Internal consistency of the MPAS and PPAS in the present study is sufficient to high (Cronbach’s alphas between .75 and .83).

#### Parenting stress

To assess parenting stress at 24 months an abbreviated, Dutch validated version of the Parenting Stress Index (Abidin 1983), the Nijmeegse Ouderlijke Stress Index, was used (NOSI-K; De Brock et al. 1992). The NOSI-K consists of 25 items which are rated from 1 (totally disagree) to 6 (totally agree), with higher scores indicating more parenting stress. Scores on all items were summed up to obtain a total score of experienced parenting stress. An example item is “Parenting this child is harder than I thought”. In the present study internal consistency of the NOSI-K for mothers and fathers was high (Cronbach’s alphas of .90 and .91 respectively).

#### Child executive functioning problems

At approximately 24 months (M = 23.7, SD = 0.77, range 23–27), child executive functioning problems were assessed with the Behavior Rating Inventory of Executive Function-Preschool Version (BRIEF-P; Gioia et al. 2003), which was developed and validated for children aged 2–5 years. In the present study, mothers completed this instrument. For 63 items, mothers were asked to indicate how often (never, sometimes, often) the mentioned behavior of their child has been a problem during the past 6 months. Scores on a Likert scale ranged from 1–3, with higher scores indicating more executive functioning problems. In the current study the BRIEF-P total score (i.e., sum score of all items) was used as in preschool-aged children executive functioning skills are still intertwined and difficult to separate into different components (Espy 2004; Isquith et al. 2004). An example item of the BRIEF-P is “Has trouble with activities or tasks that have more than one step”. Internal consistency of the BRIEF-P total score was found to be strong in the present study (Cronbach’s alpha = .93).

### Data Analyses

To examine the bivariate associations between all study variables, Pearson correlation analyses were conducted in IBM SPSS Statistics 22. In addition, a series of path models was performed in MPlus 7 (Muthén and Muthén 1998–2015) to examine the associations between parental bonding and parenting stress, and between parental bonding, parenting stress, and executive functioning. This was done for mothers and fathers separately. By using path models, multiple measurement occasions of parental bonding could be taken into account in one model predicting parenting stress. In addition, the possible mediating role of parenting stress in the association of parental bonding at 6 months and child executive functioning at 24 months could be examined within one path model, controlling for a possible mediating effect of parental bonding at 24 months. Missing values were handled by Full Information Maximum Likelihood (FIML) estimation. First, completely saturated models with all variables included were run. Covariates (gestational age, gender, parental age, parental education) were added to all the path models as predictors of the outcome variables. However, these covariates had no significant effect and did not alter the results of the models. Next, all non-significant paths (including covariates) were trimmed from the models, in line with other studies in the same research area (e.g., Östberg and Hagekull 2000), which made it possible to assess model fit. Final models with significant associations are presented and interpreted.

## Results

Correlations among, and descriptive statistics of, the study variables are presented in Table 1. Pre- and postnatal maternal bonding were moderately positively correlated with each other. Prenatal bonding had a weak negative correlation and postnatal bonding strong negative correlation with parenting stress, indicating that lower levels of bonding were associated with higher levels of parenting stress. In addition, lower levels of postnatal, but not prenatal, bonding were moderately associated with more executive functioning problems. Also, more parenting stress as experienced by the mother was strongly related to more child executive functioning problems. Fathers showed a similar pattern of correlations (see Table 1). However, no significant correlations were found between paternal bonding and child executive functioning problems. Maternal and paternal bonding were weakly to moderately correlated (over the three timepoints). In addition, a weak positive correlation was found between maternal and paternal parenting stress.

**Table 1:** Correlations between maternal (*N* = 335) and paternal bonding (*N* = 261), parenting stress, and child executive functioning

Variables	M	SD	1	2	3	4	5	6	7	8	9
1. Maternal prenatal bonding	75.46	6.30	–	.335***	.283***	−.195**	−.090	.307***	.298***	.310***	−.122
2. Maternal bonding at 6 months	83.29	5.94		–	.605***	−.497***	−.315***	.192**	.259***	.308***	−.206**
3. Maternal bonding at 24 months	80.64	6.40			–	−.635***	−.312***	.120	.229***	.238***	−.230**
4. Maternal parenting stress	42.55	13.14				–	.587***	−.042	−.102	−.122	.253**
5. EF problems	86.22	13.94					–	−.014	−.067	−.127	.226**
6. Paternal prenatal bonding	65.76	7.10						–	.554***	.533***	−.255**
7. Paternal bonding at 6 months	76.60	7.47							–	.721***	−.435***
8. Paternal bonding at 24 months	74.56	6.77								–	−.510***
9. Paternal parenting stress	42.56	13.01									–

*Note* Correlations are based on imputed date to match the sample size of the path analyses. Means and standard deviations are based on original data (*N* between 231 and 335 for mothers and *N* between 170 and 261 for fathers)

* *p* < .05, ** *p* < .01, *** *p* < .001

### Parental Bonding and Parenting Stress

Path models were used to examine the associations between parental prenatal and postnatal bonding (at 6 and 24 months) and parenting stress at 24 months. First, paths between the bonding variables at three time points as well as paths between all bonding variables and parenting stress at 24 months were included in the model. Subsequently, non-significant paths were removed. Below, we present the final models.

**Fig. 1:**
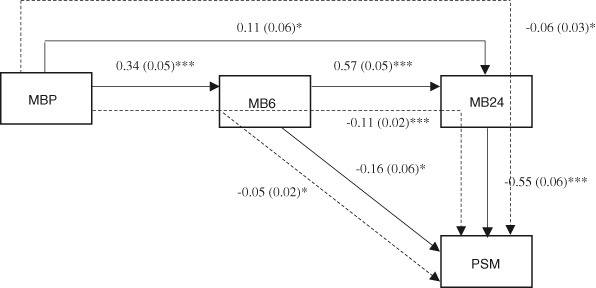
Standardized path coefficients and standard errors for the associations between maternal bonding and parenting stress. Dashed lines are used for indirect effects. * *p* < .05, ** *p* < .01, *** *p* < .001. MBP = Maternal Bonding Prenatally, MB6 = Maternal Bonding at 6 months, MB24 = Maternal Bonding at 24 months, PSM = Parenting Stress Mothers

The final path model for mothers (Fig. 1) had an excellent fit (χ^2^ = 0.163, df = 1, *p* = .686, RMSEA = .00, CFI = 1.00), and showed that prenatal bonding predicted postnatal bonding at 6 and 24 months. Postnatal bonding at 6 months also predicted bonding at 24 months. In addition, postnatal, but not prenatal, feelings of bonding were negatively related to experienced parenting stress in mothers at 24 months. Thus, higher levels of postnatal maternal bonding lead to lower levels of parenting stress. Although there was no direct path from prenatal bonding to parenting stress, the indirect paths through postnatal bonding at 6 months and at 24 months were significant, as well as the indirect longitudinal path via bonding at 6 and 24 months (see Fig. 1). This means that higher levels of prenatal bonding lead to higher levels of postnatal bonding at 6 and 24 months, which in turn lead to lower levels of parenting stress.

**Fig. 2:**
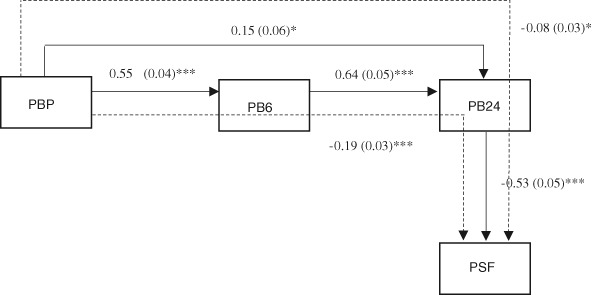
Standardized path coefficients and standard errors for the associations between paternal bonding and parenting stress. Dashed lines are used for indirect effects. * *p* < .05, ** *p* < .01, *** *p* < .001. PBP = Paternal Bonding Prenatally, PB6 = Paternal Bonding at 6 months, PB24 = Paternal Bonding at 24 months, PSF = Parenting Stress Fathers

For fathers similar patterns were found regarding the associations between paternal bonding and parenting stress (Fig. 2). Again, the final model had a good fit to the data (χ^2^ = 4.575, df = 2, *p* = .102, RMSEA = .070, CFI = .991). Paternal prenatal bonding prospectively predicted postnatal bonding at 6 and 24 months and bonding at 6 months predicted feelings of bonding at 24 months. Paternal postnatal bonding at 24 months, but not prenatal bonding or bonding at 6 months, was negatively associated with experienced parenting stress. Thus, higher levels of postnatal paternal bonding at 24 months were associated with lower levels of parenting stress in fathers. Although there was no direct path from paternal prenatal bonding to parenting stress, the indirect path through postnatal bonding at 24 months was significant, as well as the indirect longitudinal path via bonding at 6 and 24 months (see Fig. 2). This means that higher levels of prenatal bonding lead to higher levels of postnatal bonding at 6 and 24 months and parental bonding at 24 months in turn is associated with lower levels of parenting stress.

### Parental Bonding and Child Executive Functioning Problems

A mediation model was tested to examine the mediation of parenting stress and parental bonding at 24 months in the association between parental bonding at 6 months and child executive functioning. The model included a direct path from parental bonding to child executive functioning, as well as indirect paths via bonding at 24 months and via parenting stress. Furthermore, the correlation between the two mediating variables was added to the model to assess their unique contribution. Although correlation analyses (Table 1) showed no significant association between paternal bonding and child executive functioning, new recommendations for mediation analyses do not require a significant direct effect for testing indirect (i.e., mediation) effects (Hayes 2009; Rucker et al. 2011). Therefore, mediation models were tested for both mothers and fathers.

**Fig. 3:**
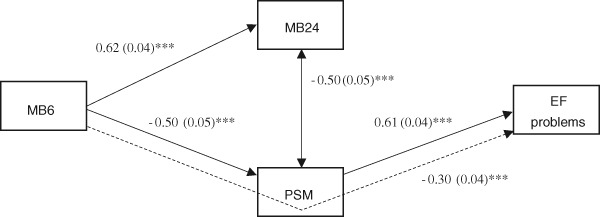
Standardized path coefficients and standard errors for the associations between maternal postnatal bonding, parenting stress, and executive functioning problems. Dashed lines are used for indirect effects. * *p* < .05, ** *p* < .01, *** *p* < .001. MB6 = Maternal Bonding at 6 months, MB24 = Maternal Bonding at 24 months, PSM = Parenting Stress Mothers, EF = Executive Functioning problems

The final model (leaving out all non-significant paths) for mothers had a good fit to the data (χ^2^ = 4.161, df = 2, *p* = .125, RMSEA = .066, CFI = .994), and results (Fig. 3) show that the (direct) association between maternal bonding at 6 months and child executive functioning problems that we observed in the correlation analyses was no longer significant in the mediation model. In addition, the path between maternal bonding at 24 months and executive functioning problems was non-significant, hence no mediation effect of postnatal bonding at 24 months was found. However, the indirect effect via parenting stress was significant, illustrating a mediation effect of parenting stress in the association between maternal bonding at 6 months and child executive functioning problems at 2 years. Higher levels of maternal bonding at 6 months lead to lower levels of parenting stress, which in turn are associated with less child executive functioning problems.

**Fig. 4:**
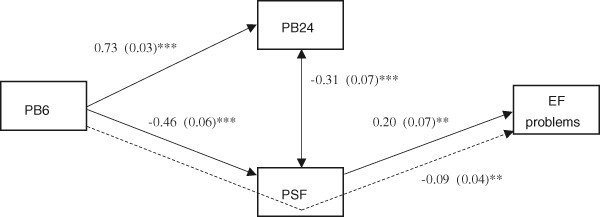
Standardized path coefficients and standard errors for the associations between paternal postnatal bonding, parenting stress, and executive functioning problems. Dashed lines are used for indirect effects. * *p* < .05, ** *p* < .01, *** *p* < .001. PB6 = Paternal Bonding at 6 months, PB24 = Paternal Bonding at 24 months, PSF = Parenting Stress Fathers, EF = Executive Functioning problems

The final model for fathers (Fig. 4) showed similar patterns of associations between bonding, parenting stress, and child executive functioning and also had a good fit to the data (*χ* ^2^ = 0.775, df = 2, *p* = .679, RMSEA = .000, CFI = 1.000). The non-significant paths between paternal bonding at 6 and 24 months and child executive functioning were removed from the model and this eliminates postnatal bonding at 24 months as a possible mediator. The indirect effect (i.e., mediation effect) of paternal bonding, via parenting stress, on child executive functioning was significant. This indicates that higher levels of paternal bonding at 6 months lead to lower levels of parenting stress, which in turn are associated with less child executive functioning problems.

## Discussion

The purpose of this study was to examine the associations between parental bonding, parenting stress, and child executive functioning. In line with our hypotheses, for both mothers and fathers, poorer prenatal bonding prospectively predicted poorer postnatal bonding, which in turn predicted more parenting stress. In addition, maternal bonding predicted child executive functioning problems, but this association was completely mediated by maternal parenting stress. For fathers, feelings of bonding also indirectly affected child executive functioning via parenting stress, however, no direct effect was found. Thus, parents with a lower quality of postnatal bonding were more vulnerable to parenting stress, which in turn was associated with more child executive functioning problems.

Our first findings offer an extension of the little previous research on parental bonding and parenting stress by showing that prenatal feelings of bonding, through its association with postnatal bonding, are already related to parenting stress experienced in toddlerhood. In addition, we found that paternal bonding is related to parenting stress experienced by fathers, which to our knowledge had not been examined before. The finding that parental (postnatal) bonding is related to parenting stress is in accordance with previous research showing that in depressed mothers a better quality of the maternal bond reduced the experience of parenting stress (Reck et al. 2016). Mothers and fathers who feel less connected to their child might experience daily stresses related to parenting more as a burden than parents who do feel a strong connection with their child. Another explanation may be that some parents are less emotionally and cognitively preoccupied with their child because of, for example, chronic stresses or preoccupations with other life goals. Therefore, they may experience raising a child as more stressful. However, these explanations are speculative and should be tested in future research. The finding that there was no direct effect (only indirect) of prenatal bonding on parenting stress might be explained by the fact that the parental bond, although relatively stable, changes over time and is also strongly affected by the birth of their child. To our knowledge, the association between parental bonding and parenting stress had not been assessed before in a community based (non-clinical) sample including both mothers and fathers. In addition, the present study adds to the literature showing that the quality of the parental bond during pregnancy is already associated with parenting stress experienced later, through its association with postnatal bonding.

Regarding the finding that parental bonding is associated with parenting stress and this in turn is related to child executive functioning development, the present study extends previous research concerning parental (mostly maternal) effects on child executive functioning. Prior studies have already linked early parenting behavior, such as parental scaffolding, sensitivity, mind-mindedness, stimulation, and control, to later child executive functioning skills (Bernier et al. 2010; Fay‐Stammbach et al. 2014). As maternal and paternal bonding comprise behavioral, affective, and cognitive components (Condon and Corkindale 1998), the present study shows that next to parental behavior, also parental feelings and cognitions are related to child executive functioning.

Parenting stress mediated the association between poor maternal and paternal postnatal bonding and child executive functioning problems. Possibly, parenting stress expresses itself in (negative) parental behavior and parent-infant interaction and in this way influences child cognitive development. However, a study by Crnic et al. (2005) found no mediating effect of parenting behavior in the association between parenting stress and child outcomes (i.e., behavior problems) and the authors argue that parenting stress also has a direct effect on child functioning. Specifically, they argued that a stressful, chaotic, and less positive environment may have a detrimental effect on children’s development even if this negative parental behavior is not directed at the child.

It has been increasingly demonstrated in previous research that early caregiving experiences have an impact on infant brain development and later child executive functioning (Fay‐Stammbach et al. 2014; Kok et al. 2015). Brain plasticity and a prolonged and rapid development of prefrontal brain regions in the first 2 years of life create a window of opportunity for early environmental experiences to affect infant brain development and a recent study indeed showed that normative variations in mother-infant interaction are associated with frontal resting EEG power, considered indicative of brain development (Bernier et al. 2016).

Another mechanism by which early parental caregiving may impact later child executive functioning is the child’s stress response system (Blair et al. 2011). Higher levels of parenting stress may create a stressful environment for children hereby influencing their own stress levels which in turn can affect neurocognitive development. Furthermore, parents provide their children with an environmental context to develop executive functioning skills. Parents who experience low levels of bonding and increased levels of parenting stress may spend less time interacting with their children and engage less in cognitively stimulating activities (e.g., playing, singing, and reading) with them. In addition, a caregiving environment dominated by high levels of parenting stress may create a chaotic and disorganized home situation in which children are not able to optimally develop executive functioning skills, whereas better family organization and greater parental warmth are associated with an increase in self-regulation and executive functioning abilities (Eisenberg et al. 2005; Schroeder and Kelley 2010; Vernon-Feagans et al. 2016). The proposed mechanisms are not mutually exclusive and may act in concert in shaping later child executive functioning. Future research is needed to examine the possible mechanisms by which early caregiving factors, such as parenting stress and bonding, affect child neurocognitive development.

### Strengths and Limitations

Some important strengths of the study are the longitudinal design already starting in pregnancy, the focus on early parental indicators and child outcomes, and the inclusion of both mothers and fathers. However, the present study also had some limitations. Although our model suggests that parental bonding affects the level of parenting stress, and this in turn (in the case of mothers) has an effect on child executive functioning, we cannot rule out that the effects also act in a different direction. In the current study, parenting stress and child executive functioning were both assessed at 24 months, making it difficult to examine directionality. Indeed, previous research, using a cross-lagged model to examine directionality, showed that parenting predicts change in child executive functioning, but child executive functioning also predicted change in parenting quality (Blair et al. 2014). However, a previous study by Reck et al. (2016) suggests that parental bonding is a stronger predictor of parenting stress rather than the other way around, confirming the direction of effect proposed in the present study. Cross-lagged models examining the association between parental bonding, parenting stress and child executive functioning could provide better insight into the direction of effects.

Another limitation of the current study is the use of self-report questionnaires. Although all questionnaires were validated and parental feelings and cognitions can only be measured via self-reports, self-reported data is always prone to response bias (e.g., social desirability). Most previous research used performance-based tests of executive functioning, providing only a momentary evaluation that is susceptible to environmental effects. In contrast, the BRIEF-P offers a measure of executive functioning in the everyday context. Parental assessments of executive functioning may be more accurate than observers’ assessments because parents know their children best and see a wide range of behaviors in different contexts. In addition, young children may underperform during executive functioning tests administered by strangers due to shyness, possibly depending on the extent to which they are familiar with interacting with non-parental caregivers. Future research could focus on the potential effect of non-parental caregivers on child executive functioning as this was not explored in the present study. However, internal consistency and test-retest stability of the BRIEF-P are accurate, and correlations with other measures of attention and behavior are consistent (Gioia et al. 2003; Isquith et al. 2005). In addition, scores on the BRIEF-P are associated with objective measures of cognitive abilities and it has predictive validity in early childhood (Clark et al. 2010).

We cannot completely rule out that maternal report on low maternal bonding, parenting stress and child executive functioning reflects a common negative or positive perceptual bias, and thus common method variance. However, a critical review of studies stating that mothers, who experience adverse psychological functioning, have distorted perceptions of their children’s problems points out that empirical evidence for this statement is questionable (Richters 1992). In addition, no direct effect of paternal bonding on mother-reported child executive functioning problems was found. While analysis of the indirect effect via paternal parenting stress revealed a significant finding, the used method deviates from classical mediation analysis practice (requiring a significant direct effect), and results should be interpreted with caution. Although maternal reports are a valid portrayal of child functioning, including father reports of child executive functioning would enable a reduction of potential reporter bias by using a cross-informant approach. In addition, future research could combine parent reported and observational data on child executive functioning and include observational data on actual parenting behavior to further strengthen the present findings.

In sum, the present study suggests that the quality of the pre- and postnatal parental bond is related to the amount of parenting stress that is experienced by both mothers and fathers. In addition, the level of parenting stress experienced is in turn related to later child executive functioning abilities. Parents who experience a lower quality bond with their child are more vulnerable to parenting stress, and their children are at risk for developing future executive functioning problems. These findings indicate a need for attention on the early parent-child relationship as experienced by the parents. Early intervention or prevention programs to improve the parental bond and reduce parenting stress could be beneficial for child cognitive development. Concepts addressing parental feelings and cognitions (e.g., bonding) should be incorporated in future research on determinants of child behavioral and (neuro)cognitive development. In addition, future longitudinal research should shed more light on the mechanisms involved in the associations of parental bonding and parenting stress with child executive functioning. This study demonstrates that, in addition to previously established relationships between parental behavior and child executive functioning, parental feelings and cognitions are important predictors of later child cognitive development as well.
